# Exploratory Study on Central Sensitization and Bioelectrical Activity of the Selected Masticatory Muscles in Subjects with Myopia

**DOI:** 10.3390/ijerph20054524

**Published:** 2023-03-03

**Authors:** Grzegorz Zieliński, Anna Matysik-Woźniak, Michał Baszczowski, Maria Rapa, Michał Ginszt, Jacek Szkutnik, Robert Rejdak, Piotr Gawda

**Affiliations:** 1 Department of Sports Medicine, Medical University of Lublin, 20-093 Lublin, Poland; 2 Department of General and Pediatric Ophthalmology, Medical University of Lublin, 20-093 Lublin, Poland; 3 Interdisciplinary Scientific Group of Sports Medicine, Department of Sports Medicine, Medical University of Lublin, 20-093 Lublin, Poland; 4 Students’ Scientific Association at the Department and Clinic of General and Pediatric Ophthalmology, Medical University of Lublin, 20-093 Lublin, Poland; 5 Department of Rehabilitation and Physiotherapy, Medical University of Lublin, 20-093 Lublin, Poland; 6 Independent Unit of Functional Masticatory Disorders, Medical University of Lublin, 20-093 Lublin, Poland

**Keywords:** myopia, sEMG, vision, optometry, masticatory muscles, temporomandibular joint, central nervous system sensitization, neurology, central sensitization inventory, oral medicine

## Abstract

Background: Myopia is one of the most common eye disorders involving abnormal focusing of light rays. The studies recognize the association between the stomatognathic and visual systems. This compound may have a neurological basis associated with disorders such as central sensitization. The main aim of this study was to evaluate the influence of central sensitization on the bioelectrical activity of selected muscles of the masticatory organ in subjects with myopia. Methods: Selected masticatory and cervical spine muscles were analyzed using an eight-channel BioEMG III electromyograph. Central sensitization was analyzed using the central sensitization inventory. Results: Statistical analysis revealed significantly higher scores on the central sensitization inventory in subjects with axial myopia compared to subjects without refractive error. Repeated positive correlations were observed in the sternocleidomastoid muscle activity and negative correlations in the digastric muscle activity during open and closed eyes in myopic subjects. Conclusions: Subjects with myopia have an increased score in the central sensitization inventory. The increase in the central sensitization inventory score is connected with the changes within the electromyographic activity of the masticatory and neck muscles. The effect of central sensitization on masticatory muscle activity in myopic subjects requires further study.

## 1. Introduction

Nociceptive pain is the sensation of pain resulting from stimuli of sufficient intensity to cause tissue damage. It occurs as a result of the activation of nociceptive pathways by peripheral stimuli [1,2]. Nociception provides neuronal feedback that enables the central nervous system to identify and avoid potentially damaging and noxious stimuli [2]. This occurs based on a withdrawal reflex from the stimulus, and when the sensation is so unpleasant that complex behavioral strategies are activated to avoid further contact with the threatening stimulus [3]. An important phenomenon that further enhances this protective function is the sensitization of the nociceptive system [3]. This is a consequence of repeated or particularly intense noxious stimuli. It results in lowering the threshold for activation of the nociceptive system and enhancing the response to subsequent stimuli with less stimulation [2,3,4]. The described phenomenon of central sensitization (CS) calls attention to the adaptive nature of the nervous system [3,4,5]. Central sensitization occurs under conditions in which the risk of further damage is high. The first to draw attention to CS was Woolf in 1983 [6]. However, a central amplification during angina pectoris was described by Sturge in 1883. He predicted the possibility of “commotion” in the central nervous system, which could “pass up from below” [7]. The International Association for the Study of Pain defines central sensitization as the increased responsiveness of nociceptive neurons in the central nervous system to their normal or subthreshold afferent input [8]. Undoubtedly, at the cellular level, CS derives from many processes that alter the functional state of nociceptive neurons [4,6,9]. These processes include increased membrane excitability, enhanced synaptic strength, and decreased inhibitory transmission (disinhibition) [3,4,6,9]. Due to the complex nature of CS, many symptoms may be related to this mechanism. The list of symptoms that may indicate CS includes fibromyalgia, chronic fatigue, irritable bowel syndrome, fatigue, sleep disturbances, depression, temporomandibular disorders, tinnitus, myofascial pain syndrome, palpitations or dizziness [4,10,11].

Myopia is one of the most common eye disorders involving abnormal focusing of light rays [12,13]. It is a condition that often begins in childhood [14]. In myopia, distant objects appear blurry to a person with a refractive error because the eye axis is too long or the cornea is too steep, causing light to be focused in front of the retina [13]. Axial myopia is the most common type of myopia [15,16]. It is assumed that for every millimeter of the overly long eyeball, there are three diopters of myopia [15]. As axial myopia depends on the length of the eyeball, the refractive error increases rapidly in adolescence [15]. According to the guidelines of The International Myopia Institute, low myopia is defined as a condition in which the spherical equivalent refractive error of an eye is ≤−0.5 and >−6.00 Dioptres (D), whereas high myopia is defined as a condition in which the spherical equivalent refractive error of an eye is ≤−6.00 D when ocular accommodation is relaxed [17]. In many industrialized countries, myopia occurs in more than 50% of the population and is expected to increase in the coming years [18]. There is no single cause of myopia. It can be suggested that myopia is the result of an interaction between genetic predisposition and environmental exposures [12].

The studies recognize the association between the stomatognathic and visual systems [19,20,21,22]. However, it is still difficult to state clearly which anatomical pathway establishes this connection. It is yet unknown whether it is the link between the stomatognathic system and the visual system related to the vestibulo-ocular reflex (VOR) [20,21,22,23,24], or a muscle--fascial connection [22,23,24], or a connection related to the CS [23]. In the hypothesis related to the CS-level connection, it is extremely difficult to answer whether it is secondary or primary to the refractive error. Blurred vision can be hypothesized to be linked to threatening stimuli [23]. For example, individuals with myopia are more likely to suffer from dry eye syndrome [25], and its recurrence may be associated with CS [26]. Hypothetically, other symptoms associated with CS (headaches, neck and shoulder muscle bioelectrical activity, tinnitus, temporomandibular joint pain [4,27,28,29]) may influence the visual system.

To the best of our knowledge, it has not yet been determined whether patients with myopia have central sensitization-related symptoms and how it affects masticatory muscle activity in myopic subjects compared to subjects without refractive error. The main aim of this study was to evaluate the influence of myopia on bioelectrical activity of selected muscles of the masticatory organ and central sensitization. The secondary aim was to compare the central sensitization inventory (CSI) results between subjects with myopia and subjects without refractive error. The main hypothesis was that a correlation between selected masticatory muscle bioelectrical activity and CSI scores would be observed. The side hypothesis was that people with myopia would have higher CSI scores compared to people without refractive errors.

## 2. Materials and Methods

### 2.1. Study Population

The study was approved by the local Bioethics Committee of the Medical University of Lublin (approval number KE-0254/229/2020). It was conducted in accordance with the principles of the Declaration of Helsinki and with the Good Clinical Practice guide. All subjects gave written informed consent. They were informed about the aims of the study and could withdraw at any time.

A total of 201 people were enrolled in the study. The following exclusion criteria were used: hyperopia; astigmatism; ocular diseases; diseases of the anterior segment of the eyeball; optic nerve diseases; class II and III of the bite according to Angle’s classification; open bite; crossbite; any form of TMDs found according to the two-axis Research Diagnostic Criteria for Temporomandibular Disorders (RDC/TMD); headache and/or orofacial pain on the examination day; of four support zones in dental arches; lack of more than four teeth within both dental arches; carious or damaged dental tissues; any oral inflammation; any periodontal pathology; any pathology or asymmetry in craniofacial structures; undergoing an orthodontic treatment; possession of dental prostheses (regardless of type); craniofacial trauma; Botox therapy; neoplastic diseases (regardless of type and location); mental and neurological disorders; head and neck injuries within the last 6 months before the study; neck and shoulder pain regardless of etiology within the last 6 months; previous head and neck surgical treatment within the last 6 months before the examination; and pregnancy.

The inclusion criteria used in the study were: age range 18–30 years; axial myopia based on clinical examination or no refractive error; normal intraocular pressure; four zones of arch support; complete dentition; and occlusion I according to the Angel classification.

The exclusion and inclusion criteria analysis was based on three examinations. The first was a clinical study based on the RDC/TMD [30]. It was performed by a dentist with a specialization in dental prosthetics (author J. S.). Palpation and functional examination of the cervical spine muscles and muscles of the masticatory organ and related structures were performed by a physiotherapist (author G. Z.). In the third stage, an ophthalmological examination was conducted. The study was performed according to the previously published methodology [22].

Based on the exclusion criteria, 78 subjects were qualified for the study. They were divided into a study group (subjects with myopia) and a control group (healthy subjects without refractive error).

### 2.2. Study Protocol

#### 2.2.1. Assessment of the Bioelectrical Muscle Activity

Surface electromyography (sEMG) testing was performed according to a standardized and reproducible protocol [21,22,24,31,32,33,34]. The subjects were seated in a standardized position on a dental chair. The headrest of the chair was adjusted for each person to maintain their natural cervical position. The examination was performed without visual correction for subjects with refractive error. The examination took place in the morning.

The skin of the subjects was cleansed with 90% ethanol. It was hairless. A physiotherapist experienced in sEMG placed the disposable electrodes in alignment with the muscle fibers. Two electrodes were placed symmetrically on each side of the muscle belly using the surface EMG method for a non-invasive assessment of muscles (SENIAM) project [35]. The reference electrode was placed on the forehead [21,22,24,31,32,33,34].

An 8-channel BioEMG III electromyograph, compatible with the BioPAK measurement system (BioResearch Associates, Inc., Milwaukee, WI, USA) and disposable electrodes (Ag/AgCl with a diameter of 30 mm and a conductive surface of 16 mm-SORIMEX, Torun, Poland) were used for analysis. Four muscle segments were analyzed:

- The anterior part of the temporalis muscle (TA);
- The superficial part of the masseter muscle (MM);
- The middle part of the sternocleidomastoid muscle belly (SCM);
- The anterior belly of the digastric muscle (DA).

Electromyographic activity was recorded in four conditions:

- At rest (10 s);
- During maximal voluntary clenching in the intercuspal position (as hard as possible; 3 × 3 s, 2 s rest between);
- During maximal voluntary clenching on dental rollers (as hard as possible; 3 × 3 s, 2 s rest between);
- During maximal mouth opening (as wide as possible; 3 × 3 s, 2 s rest between).

Averaged records from three trials were included in the analysis. Automatic processing of the electromyographic signal based on the calculation of root mean square (RMS) values in the BioPAK software produced mean values, which were then used to analyze muscle activity [21,24,31]. The study was conducted with both eyes open (subjects looked ahead) and eyes closed, with a 5 min interval between trials. The initial trial was randomly selected. Both tests were performed without corrective lenses and eyeglasses [20,21,24]. Before each test, an interference test was performed using the BioPAK Measurement System, and after the test, BioPAK Noise Tests (BioResearch Associates, Inc. Milwaukee, WI, USA) were performed. In addition, all electromyographic signals were visually inspected before each RMS calculation to obtain RMS only during steady-state periods. Signal analysis was performed by the same physiotherapist (author G. Z.).

#### 2.2.2. Ophthalmological Examination

At the very first step, a refractive error test was conducted using the Topcon KR-800 autokeratorefractometer (Topcon Co. Tokyo, Japan). The test was carried out without mydriatics.

Next, visual acuity (VA) of emmetropic subjects and Best Corrected Visual Acuity (BCVA) of myopic subjects were checked. VA and BCVA were analyzed separately for the right and left eye. The ETDRS chart was used to test visual acuity [36]. The examination of visual acuity with the ETDRS chart was performed from a distance of 4 m.

An IOL Master 500 (Carl Zeiss Meditec, Jena, Germany) was used for the analysis of eyeball length, and a Tono-Pen XL (Medtronic Solan, FL, USA) was used for intraocular pressure. The structures of the eyeball were evaluated using a slit lamp. It was conducted by an experienced ophthalmologist (author A. M-W.). The study was performed according to previously published methodology [22].

#### 2.2.3. Assessment of the Central Sensitization Inventory

The Central Sensitization Inventory was developed in 2011 [37]. The main purpose of the questionnaire is to assess patients with central sensitization syndrome [11,37,38,39]. The Polish language version of the CSI was published in 2019 [11], and the psychometric version was validated [39]. The Polish version of the CSI demonstrated excellent internal consistency (Cronbach’s alpha = 0.933) and test–retest reliability (Intraclass Correlation Coefficients = 0.96) [39]. The questionnaire for the present study was downloaded from https://www.pridedallas.com/questionnaires/ accessed on 14 December 2022.

CSI is composed of 2 parts. The first part, called part A, consists of 25 items. The questionnaire is designed to be self-completed. It deals with symptoms related to central sensitization (e.g., “I feel tired and unrefreshed when I wake from sleeping”, “My muscles feel stiff and achy”, “I have anxiety attacks”, “I grind or clench my teeth”). Each sub-item is scored from 0 to 4 according to a Likert scale. Each question can be answered as follows: never (0 points), rarely (1 point), sometimes (2 points), often (3 points), or always (4 points). The maximum score is 100. Higher scores are associated with a greater degree of self-reported symptoms [11,37,38,39]. Part B is not scored. It gives information about the course of the disease. The respondent indicates whether he/she has been diagnosed with any of the listed diseases along with the year of diagnosis (restless leg syndrome, chronic fatigue syndrome, fibromyalgia, temporomandibular joint disorder, tension headaches/migraines, irritable bowel syndrome, multiple chemical sensitivities, neck injury, anxiety/panic attacks, or depression) [11,37,38,39].

#### 2.2.4. Statistical Analysis

These calculations were performed using Statistica™ version 13.3 (TIBCO Software Inc., Palo Alto, CA, USA). First, the normality of the distribution of variables was verified using the Shapiro–Wilk test and the Kolmogorov–Smirnov test (with Lillierfors correction). Depending on the distribution, the Student *t*-test (t) or Mann–Whitney U test (z) was used. Effect sizes were determined for the *t*-test using the Cohen d method and interpreted as small (0.2), medium (0.5), and large (0.8) effect sizes [40,41].

The Spearman Rho test was used because the distribution was not normal for CSI, and for the bioelectrical activity of the selected masticatory and cervical muscles. The test’s scale began at −1 (perfect negative monotonic association) and finished at +1 (perfect positive monotonic association). A correlation was considered large for values greater than 0.5 and moderate for values between 0.3 and 0.5 [42].

An analysis of power was conducted using G*Power 3.1 (Heinrich-Heine-Universität Düsseldorf, Düsseldorf, Germany) [43]. The calculations indicated that a sample size of 17 participants would be sufficient to notice a significant differences between matched pairs (*t*-test) with an α value of 0.05, a power value of 0.90, and an estimated medium effect size of 0.75. Statistical significance was set at *p* ≤ 0.05. The significance level was reported with a 95% confidence interval (95%CI). LabPlot (Version 2.9.0, KDE, Berlin, Germany) was used to graphically represent the data.

## 3. Results

Table 1 shows a comparison of the groups in terms of size and anatomical features. Statistically, the groups differed only in refractive error and axial length.

**Figure 1:**
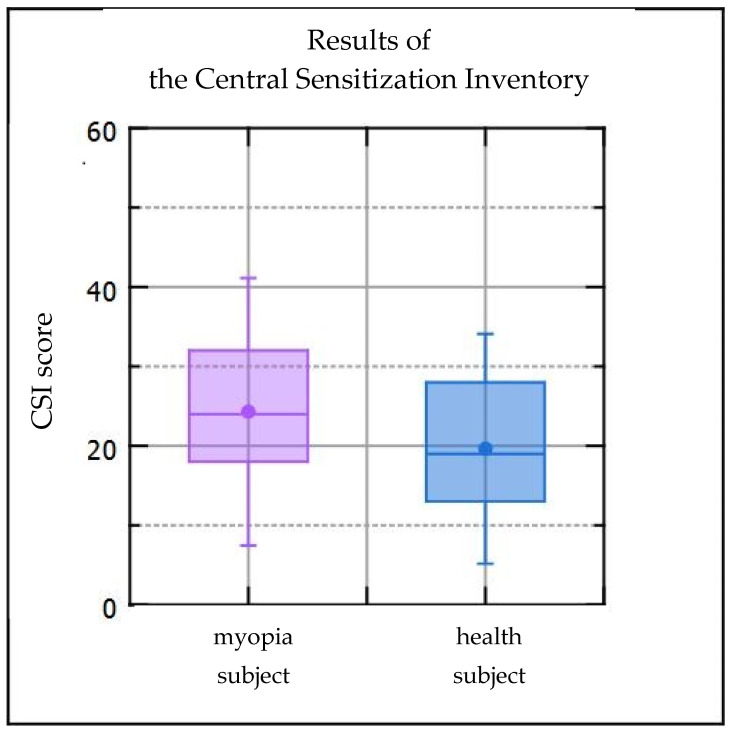
Graphical representation of CSI scores in myopic subjects and healthy subjects. CSI—the central sensitization inventory.

Statistical analysis revealed significantly higher scores on the central sensitization inventory in subjects with axial myopia compared to subjects without refractive errors (Table 2, Figure 1).

In the myopic group, statistical analysis between the CSI score and muscle activity showed significant positive correlations in resting SCM-R muscle activity and in DA-R activity during clenching on dental cotton rollers in the open eye test. In the same group, negative correlations were observed within DA-R and DA-L muscle activity during maximum mouth opening in the open eyes test (Table 3). In the healthy subjects, positive correlations were observed for DA-R during clenching in the intercuspal position and clenching on dental cotton rollers in the open eyes test (Table 3).

Statistical analysis between the CSI score and muscle activity showed significant positive correlations in resting SCM-R activity in the myopic group in the closed eyes test. In the same group, negative correlations were observed on DA-R and DA-L muscle activity during maximum mouth opening in the closed eyes test (Table 4). In the healthy subjects, positive correlations were observed for the SCM-R, SCM-L, and DA-R muscle groups during clenching in the intercuspal position in the closed eyes test (Table 4).

**Figure 2:**
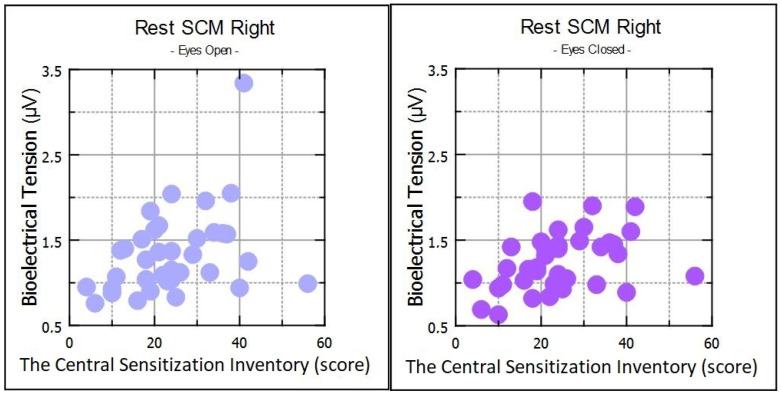
Scatter plot for SCM-R in rest for open eyes and closed eyes.

**Figure 3:**
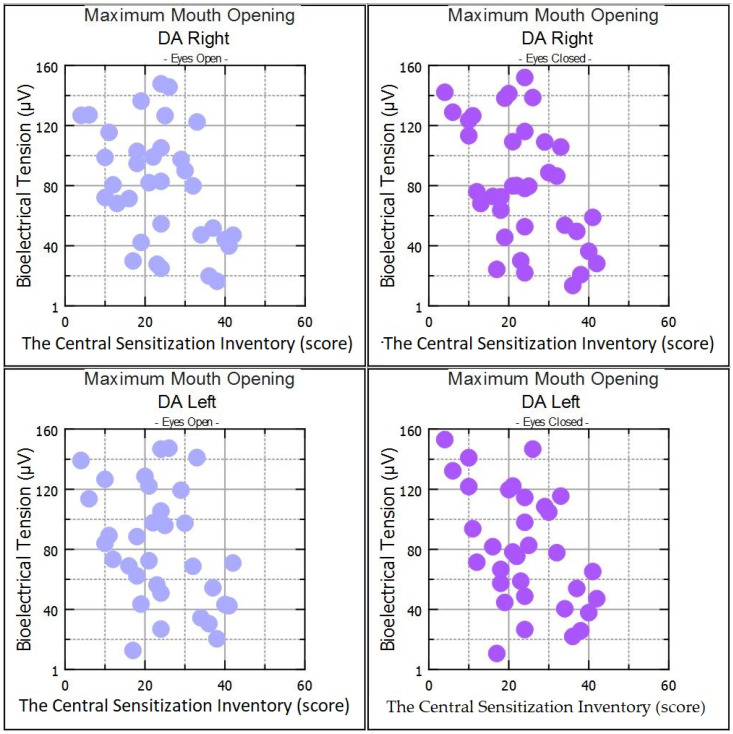
Scatter plot for DA-R and DA-L in maximum mouth opening for open eyes and closed eyes.

Repeated correlations were observed only within SCM-R in the resting mandibular position in the open and closed eyes tests (Figure 2), and within DA-R and DA-L during maximum mouth opening (Figure 3, Table 3 and Table 4) in myopic subjects. Correlations were also observed in DA in both tests on clenching dental cotton rollers in healthy subjects (Table 3 and Table 4).

## 4. Discussion

**Figure 4:**
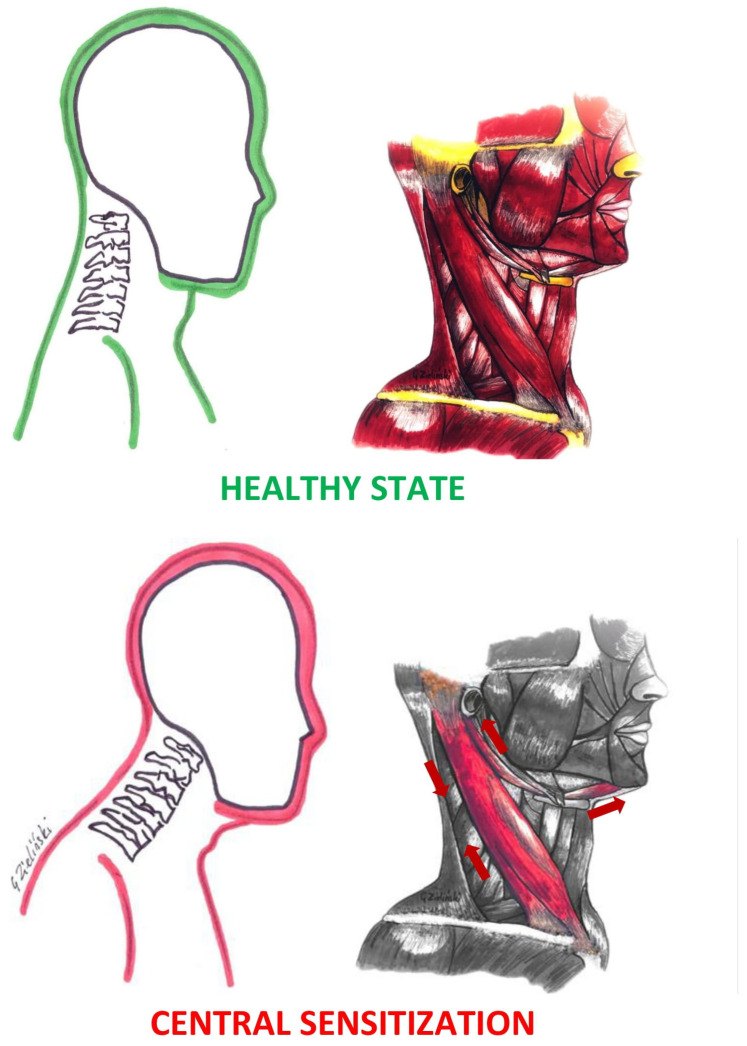
Hypothetical presentation of changes in muscle activity with increasing central sensitization inventory score.

The main objective of this study was accomplished. The association of central sensitization on bioelectrical activity of selected muscles of the masticatory organ in subjects with myopia has been evaluated. A correlation linked to SCM and DA muscles was observed in both tests. The correlation recurred despite the change of visual stimulus (eyes open vs. closed) in subjects with myopia. In subjects without refractive errors, correlations were observed during the closed eyes test. The results obtained indicate a constant increase in the central sensitization inventory score in subjects with myopia. The main hypothesis and the side hypothesis were confirmed. DA and SCM muscle activity, along with higher CSI values, indicate possible changes in the cervical region and head position. Positive SCM correlations may indicate muscle shortening, and negative DA correlations may indicate muscle stretching (Figure 4).

Our analysis is unable to determine the cause and effect relationship between CS and myopia. Therefore, further hypotheses are related to the possible explanation of the obtained results. Central sensitization represents an over-active circuit function in nociceptive pathways and the abnormal state of nociceptive systems [3,44]. It is important to consider whether blurred vision will be treated by the brain like a nociceptive stimulus. In patients with myopia, increased bioelectrical activity of the craniofacial muscles (test without visual correction) is observable with open eyes, while with closed eyes, there is a decrease in bioelectrical activity [20,21,24]. This phenomenon has not been observed in subjects without refractive errors [45]. It was also observed that with a greater correction of refractive error, the resting temporal muscle activity increases [31]. The results obtained in several studies [20,21,24,31,45] suggest plasticity of the muscle response in individuals with myopia. These changes may be related to the VOR [46]. A reflex that further assists in achieving stabilization of the visual target and image on the retina is the carotid-eye reflex, which works in conjunction with the VOR. Information is processed by three sources: the visual system via the eyes, proprioceptive receptors, and vestibular receptors in the inner ear [23]. The vestibular complex receives afferent information from other parts of the central nervous system—such as the reticular formation—parts of the midbrain nuclei, the cerebellum, and especially the spinal cord [47]. The trigeminal nuclei are probably involved in the transmission of information [23]. Due to their size, the trigeminal nuclei mediate impulses from all cranial nerves [46].

The change in the activation patterns of the masticatory and cervical muscles due to blurred vision (no correction or insufficient correction) can be perceived as a threat to the body. One of many, it may lead to the disruption of muscle–fascial homeostasis. Muscle nociceptors are free nerve endings connected to the central nervous system [48]. Two chemical stimuli play a particularly significant role in muscle pain. The first one induces a decrease in tissue pH, and the second is the release of adenosine triphosphate (ATP) [48]. Any damage within muscle cells or increase in their membrane permeability is accompanied by the release of ATP from the cells [49]. It is worth noting that ATP is considered by some as a general pain stimulus [48]. Abnormal muscle activity caused by blurred vision (in the VOR pathway described above [23]) may hypothetically cause a sudden increase in ATP demand, and its decrease may result in changes in pH value and related nociceptive activation.

Another hypothetical explanation for the above-mentioned relationship would be the occurrence of biomechanical or psychosomatic changes. Short-sighted individuals often compensate for vision problems by leaning forward or turning their heads from side to side, which leads to increased bioelectrical activity in the pectoralis muscles, descending fibers of the trapezius, sternoclavicular muscles, suboccipital muscles, and scapular elevation [24,50]. This may be caused by several factors. Short-sighted people often have higher IQ levels, which are associated with longer time spent studying and less time spent outdoors [51]. The amount of time spent studying may be related to poor posture and stress (related to wanting to master the material). It has been proven that mental challenges are associated with anxiety and biomechanical changes in the form of forwarding head tilt [52,53,54]. Musculoskeletal changes may develop as a result of mental changes. It has been observed that stress may play a role in myopia [55]. Correlations have also been noted between myopia and mental health, especially anxiety [56]. Another study found that myopia was associated with anxiety and anxiety scores. The higher the degree of myopia, the higher the anxiety score [57]. The described position changes may also lead to overloading the aforementioned muscles and nociceptive changes in ATP release and pH. This may also explain the obtained higher CSI values in subjects with myopia.

It remains unclear whether central sensitization may be the primary cause or a secondary effect of refractive error. The effect of CS on selected masticatory muscle activity in healthy subjects is not fully understood. Future research should focus on investigating this phenomenon, particularly in different age groups (e.g., children) and other races, as myopia is connected with race and ethnicity [58,59]. In contrast, prospective studies on children and adolescents could clarify whether myopia may result from central sensitization.

The study presented here has several limitations. We point out that the most objective markers of CS, such as functional neuroimaging, are still under development. They were not used in this study. Quantitative sensory testing mostly involves testing skin sensitivity, rather than the deep pain often experienced by patients with suspected CS. Future research on CS using functional neuroimaging should be considered. Another limitation of this study is the high risk of a selection bias that can lead to interpretation bias due to the use of CSI on healthy individuals. The next limitation is that the CSI has good clinical measurement properties (e.g., validity and reliability), but a study by Coronado et al. has challenged their construct validity as this questionnaire was associated with widespread pain sensitivity. Both showed a stronger association with psychological measures than with markers for central sensitization [60].

## 5. Conclusions

- Subjects with myopia have an increased score in the central sensitization inventory.
- The increase in the central sensitization inventory score is connected with the changes in the electromyographic activity of the masticatory and neck muscles.
- The effect of central sensitization on masticatory muscle activity in myopic subjects requires further studies.

## Figures and Tables

**Table 1:** Presentation of groups.

		Myopic Group		Healthy Group		Test		*p*
		Mean	SD	Mean	SD			
*n*		37.00		41.00				
Refractive Error (Diopters)	R	−2.50	−1.50	-	-			
	L	−2.50	−1.50	-	-			
Best Corrected Visual Acuity	R	1.0		-				
	L	1.0		-				
Visual Acuity	R	-		1.0				
	L	-		1.0				
Intraocular Pressure (mmHg)	R	14.52	4.81	15.87	3.49	z	−0.98	0.33
	L	15.74	3.25	14.70	4.00	z	0.65	0.52
Axial Length (mm)	R	24.38	0.63	23.59	0.63	z	3.51	0.00 * ES = 0.25
	L	24.38	0.66	23.59	0.62	z	3.49	0.00 * ES = 0.25
Sex	F	24		28		z	0.31	0.75
	M	13		13				
Age (years)		22.92	1.69	23.19	1.06	z	−0.74	0.46
Height (cm)		172.62	9.64	171.02	8.17	z	0.34	0.73
Weight (kg)		68.19	17.33	65.50	12.04	z	0.36	0.72
Body Mass Index		22.59	3.67	22.30	3.21	z	0.00	1.00
Mandibular Range Of Motion (mm)	Abduction Without Pain	51.16	7.34	50.86	5.44	t	0.21	0.83
	Active Abduction	51.73	7.01	52.00	5.77	t	−0.19	0.85
	Passive Abduction	54.05	7.03	54.24	6.15	t	−0.12	0.90
	Mandibular Movement to The Right	9.16	1.86	9.17	2.67	z	−0.39	0.70
	Mandibular Movement to The Left	9.81	1.98	9.88	2.46	z	−0.54	0.59
	Protrusion	8.86	2.30	8.60	2.49	z	0.69	0.49

F—female; M—male; R—right side; L—left side; * significant difference.

**Table 2:** Summary of ICS scores in myopic subjects and healthy subjects.

	Myopic Subjects		Healthy Subjects		Test		*p*	95% IC
	Mean	SD	Mean	SD				
CSI	24.30	11.22	19.57	9.54	t	2.02	0.04 * ES = 0.45	0.05, 9.41

CSI—the central sensitization inventory; ES—effect size; * significant difference.

**Table 3:** Summary of myopic subjects’ and healthy subjects’ results of the open eye test.

		Myopic Subjects						Healthy Subjects					
		Mean	SD	R	t(N-2)	*p*	95% IC	Mean	SD	R	t(N-2)	*p*	95% IC
Rest	TA-R	2.30	1.87	0.08	0.46	0.65	−0.25, 0.39	2.50	1.92	0.05	0.31	0.76	−0.26, 0.35
	TA-L	2.36	1.34	0.21	1.26	0.22	−1.22, 0.50	2.20	1.53	0.06	0.35	0.73	−0.25, 0.36
	MM- R	2.36	1.56	0.12	0.70	0.49	−0.21, 0.43	2.53	1.87	0.01	0.03	0.97	−0.30, 0.31
	MM-L	2.42	1.65	−0.06	−0.37	0.72	−0.38, 0.27	2.14	0.96	0.00	0.01	0.99	−0.31, 0.31
	SCM-R	1.33	0.49	0.38	2.41	0.02 *	0.60, 0.63	1.17	0.34	0.15	0.93	0.36	−0.17, 0.44
	SCM-L	1.47	0.51	0.07	0.42	0.68	−0.26, 0.39	1.24	0.31	0.09	0.55	0.59	−0.22, 0.39
	DA-R	1.92	1.09	0.15	0.90	0.37	−0.18, 0.45	1.75	0.88	0.20	1.24	0.22	−0.11, 0.48
	DA-L	1.93	1.14	−0.07	−0.39	0.70	−0.39, 0.26	1.68	0.81	0.02	0.09	0.93	−0.29, 0.33
Clenching in the intercuspal position	TA-R	151.45	73.61	−0.15	−0.89	0.38	−0.45, 0.18	149.95	82.19	0.13	0.80	0.43	−0.19, 0.42
	TA-L	155.35	81.38	−0.08	−0.49	0.63	0.39, 025	139.57	71.21	0.04	0.22	0.83	−0.27, 0.34
	MM- R	186.89	133.93	−0.10	−0.57	0.57	−0.41, 0.23	169.12	98.21	0.04	0.25	0.80	−0.27, 0.34
	MM-L	170.74	125.81	−0.06	−0.36	0.72	−0.38, 0.27	162.76	104.02	0.08	0.51	0.61	−0.23, 0.38
	SCM-R	14.05	11.34	0.08	0.49	0.63	−0.25, 0,39	11.22	7.39	0.19	1.19	0.24	−0.12, 0.47
	SCM-L	13.82	10.41	0.18	1.08	0.29	−0.15, 0.48	13.08	12.40	0.15	0.94	0.35	−0.17, 0.44
	DA-R	24.08	17.15	0.24	1.46	0.15	0.09, 0,52	20.64	12.01	0.33	2.10	0.04 *	0.03, 0.58
	DA-L	26.11	21.97	0.11	0.62	0.54	−0.22, 0.42	19.83	12.16	0.21	1.31	0.20	−0.10, 0.49
Clenching on dental cotton rollers	TA-R	134.69	61.21	−0.05	−0.28	0.78	−0.37, 0.28	140.94	79.85	0.22	1.41	0.17	−0.09, 0.49
	TA-L	137.20	68.47	0.03	0.16	0.87	−0.30, 0.35	135.89	61.96	0.20	1.26	0.21	−0.11, 0.49
	MM- R	191.50	107.30	0.03	0.15	0.88	−0.30, 0.35	183.48	85.64	0.22	1.40	0.17	−0.09, 0.49
	MM-L	179.62	107.82	−0.05	−0.31	0.76	−0.37, 0.28	183.47	86.04	0.23	1.49	0.14	−0.08, 0.50
	SCM-R	15.21	10.95	0.23	1.38	0.18	−0.10, 0.52	13.09	7.02	0.29	1.90	0.07	−0.02, 0.55
	SCM-L	15.34	8.98	0.30	1.87	0.07	−0.03, 0.57	14.95	11.35	0.28	1.82	0.08	−0.03, 0.54
	DA-R	25.54	15.03	0.43	2.75	0.01 *	0.12, 0.66	21.17	9.22	0.37	2.39	0.02 *	0.07, 0.61
	DA-L	27.54	20.12	0.21	1.28	0.21	−1.12, 0.50	21.64	9.77	0.15	0.92	0.36	−0.17, 0.44
Maximum mouth opening	TA-R	10.45	11.90	0.14	0.85	0.40	−0.19, 0.44	5.90	5.09	−0.14	−0.91	0.37	−0.43, 0.18
	TA-L	9.25	9.08	0.19	1.12	0.27	−0.14, 0.48	6.10	5.72	−0.12	−0.76	0.45	−0.41, 0.19
	MM- R	14.87	17.88	0.15	0.90	0.37	−0.18, 0.45	9.58	11.55	−0.27	−1.74	0.09	−0.53, 0.04
	MM-L	12.64	12.69	0.13	0.77	0.45	−0.20, 0.44	8.14	6.80	−0.25	−1.61	0.12	−0.52, 0.06
	SCM-R	16.15	18.54	0.21	1.27	0.21	−1.12, 0.50	8.61	6.52	−0.12	−0.75	0.46	−0.41, 0.19
	SCM-L	17.79	23.70	0.17	0.99	0.33	−0.16, 0.47	9.84	13.09	−0.06	−0.36	0.72	−0.36, 0.25
	DA-R	84.91	41.96	−0.36	−2.28	0.03 *	−0.61, −0.04	60.32	38.28	−0.10	−0.59	0.56	−0.40, 0.21
	DA-L	88.43	46.72	−0.35	−2.21	0.03 *	−0.61, −0.03	60.71	41.81	−0.02	−0.10	0.92	−0.33, 0.29

TA—the anterior part of the temporalis muscle; MM—the superficial part of the masseter muscle; SCM—the middle part of the sternocleidomastoid muscle; DA—the anterior belly of the digastric muscle; R—right side; L—left side; * significant difference.

**Table 4:** Summary of myopia subjects’ and health subjects’ results of the closed eyes test.

		Myopic Subjects						Healthy Subjects					
		Mean	SD	R	t(N-2)	*p*	95% IC	Mean	SD	R	t(N-2)	*p*	95% IC
Rest	TA-R	2.02	1.26	0.08	0.47	0.64	−0.25, 0.39	1.96	1.29	0.13	0.82	0.41	−0.19, 0.42
	TA-L	2.22	1.54	0.05	0.27	0.79	−0.28, 0.37	1.78	1.03	0.17	1.05	0.30	−0.15, 0.45
	MM- R	2.23	1.37	0.14	0.80	0.43	−0.19, 0.44	2.34	1.50	−0.17	−1.09	0.28	−0.45, 0.15
	MM-L	2.33	2.01	0.02	0.14	0.89	−0.31, 0.34	2.23	1.51	0.12	0.78	0.44	−0.19, 0.41
	SCM-R	1.24	0.33	0.40	2.62	0.01 *	0.09, 0.64	1.33	0.59	0.13	0.82	0.42	−0.19, 0.42
	SCM-L	1.42	0.46	0.27	1.68	0.10	−0.06, 0.55	1.39	0.58	0.00	0.00	1.00	−0.31, 0.31
	DA-R	1.70	0.73	−0.28	−1.70	0.10	−0.55, 0.05	1.69	0.62	0.10	0.58	0.56	−0.21, 0.40
	DA-L	1.72	0.76	−0.33	−2.01	0.05	−0.59, −0.01	1.57	0.51	0.10	0.60	0.55	−0.21, 0.40
Clenching in the intercuspal position	TA-R	135.21	67.91	−0.15	−0.87	0.39	−0.45, 0.18	135.73	67.68	0.10	0.61	0.54	−0.21, 0.40
	TA-L	140.55	76.46	−0.06	−0.38	0.70	−0.38, 0.27	131.28	63.63	0.01	0.06	0.95	−0.30, 0.32
	MM- R	162.55	120.38	−0.11	−0.63	0.53	−0.42, 0.22	143.93	89.67	0.15	0.92	0.37	−0.17, 0.44
	MM-L	151.62	115.81	−0.06	−0.35	0.73	−0.38, 0.27	139.18	85.60	0.05	0.34	0.74	−0.26, 0.35
	SCM-R	11.31	9.25	0.07	0.40	0.70	−0.26, 0.39	9.38	6.26	0.18	1.12	0.27	−0.14, 0.46
	SCM-L	11.09	7.70	0.15	0.88	0.38	−0.18, 0.45	10.51	8.77	0.21	1.35	0.18	−0.10, 0.49
	DA-R	20.78	13.44	0.18	1.08	0.29	−0.15, 0.48	17.63	11.22	0.29	1.84	0.07	−0.02, 0.55
	DA-L	22.19	18.52	0.09	0.54	0.60	−0.24, 0.40	16.61	11.33	0.21	1.31	0.20	−0.10, 0.49
Clenching on dental cotton rollers	TA-R	135.04	56.62	−0.06	−0.36	0.72	−0.38, 0.27	140.88	76.42	0.17	1.10	0.28	−0.15, 0.45
	TA-L	137.40	63.16	−0.06	−0.36	0.72	−0.38, 0.27	136.83	62.15	0.20	1.27	0.21	−0.11, 0.48
	MM- R	184.33	97.21	0.06	0.38	0.71	−0.27, 0.38	173.64	85.50	0.26	1.68	0.10	−0.05, 0.53
	MM-L	177.26	105.41	0.02	0.09	0.93	−0.31, 0.34	174.21	82.26	0.25	1.62	0.11	−0.06, 0.52
	SCM-R	13.93	9.79	0.19	1.15	0.26	−0.14, 0.48	12.38	7.26	0.39	2.67	0.01 *	0.09, 0.62
	SCM-L	14.48	8.75	0.18	1.11	0.27	−0.15, 0.48	14.10	10.75	0.32	2.07	0.04 *	0.01, 0.57
	DA-R	23.74	12.74	0.29	1.78	0.08	−0.04, 0.56	19.28	8.46	0.36	2.35	0.02 *	0.06, 0.60
	DA-L	25.48	20.35	0.23	1.37	0.18	−0.10, 0.52	18.83	9.50	0.26	1.63	0.11	−0.05, 0.53
Maximum mouth opening	TA-R	9.28	8.19	0.08	0.50	0.62	−0.25, 0.39	5.07	3.01	−0.21	−1.33	0.19	−0.49, 0.10
	TA-L	8.77	7.84	0.15	0.92	0.36	−0.18, 0.45	4.79	3.34	−0.23	−1.50	0.14	−0.50, 0.08
	MM- R	13.86	16.96	0.03	0.19	0.85	−0.30, 0.35	11.01	16.35	−0.09	−0.54	0.59	−0.39, 0.22
	MM-L	12.15	10.99	0.04	0.24	0.81	−0.29, 0.36	6.93	4.46	−0.11	−0.66	0.51	−0.40, 0.20
	SCM-R	15.64	17.91	0.07	0.42	0.68	−0.26, 0.39	8.72	8.04	−0.16	−0.99	0.33	−0.45, 0.16
	SCM-L	18.23	24.95	0.06	0.36	0.72	−0.27, 0.38	9.63	11.19	−0.07	−0.42	0.68	−0.37, 0.24
	DA-R	81.52	40.20	−0.43	−2.75	0.01 *	−0.66, −0.12	65.44	44.87	−0.12	−0.73	0.47	−0.41, 0.19
	DA-L	88.43	46.72	−0.35	−2.21	0.03 *	−0.61, −0.03	60.71	41.81	−0.02	−0.10	0.92	−0.33, 0.29

TA—the anterior part of the temporalis muscle; MM—the superficial part of the masseter muscle; SCM—the middle part of the sternocleidomastoid muscle; DA—the anterior belly of the digastric muscle; R—right side; L—left side; * significant difference.

## Data Availability

The datasets generated during and/or analyzed during the current study are available from the corresponding author on reasonable request.
